# Sex–Gender Disparities in Cardiovascular Diseases: The Effects of Estrogen on eNOS, Lipid Profile, and NFATs During Catecholamine Stress

**DOI:** 10.3389/fcvm.2021.639946

**Published:** 2021-02-12

**Authors:** Marie Louise Ndzie Noah, Gabriel Komla Adzika, Richard Mprah, Adebayo Oluwafemi Adekunle, Joseph Adu-Amankwaah, Hong Sun

**Affiliations:** Department of Physiology, Xuzhou Medical University, Xuzhou, China

**Keywords:** catecholamine stress, β-adrenergic receptors, estrogen, eNOS, lipid profile, NFATs, cardiovascular diseases

## Abstract

Cardiovascular diseases (CVDs) characterized by sex–gender differences remain a leading cause of death globally. Hence, it is imperative to understand the underlying mechanisms of CVDs pathogenesis and the possible factors influencing the sex–gender disparities in clinical demographics. Attempts to elucidate the underlying mechanisms over the recent decades have suggested the mechanistic roles of estrogen in modulating cardioprotective and immunoregulatory effect as a factor for the observed differences in the incidence of CVDs among premenopausal and post-menopausal women and men. This review from a pathomechanical perspective aims at illustrating the roles of estrogen (E2) in the modulation of stimuli signaling in the heart during chronic catecholamine stress (CCS). The probable mechanism employed by E2 to decrease the incidence of hypertension, coronary heart disease, and pathological cardiac hypertrophy in premenopausal women are discussed. Initially, signaling via estrogen receptors and β-adrenergic receptors (βARs) during physiological state and CCS were summarized. By reconciling the impact of estrogen deficiency and hyperstimulation of βARs, the discussions were centered on their implications in disruption of nitric oxide synthesis, dysregulation of lipid profiles, and upregulation of nuclear factor of activated T cells, which induces the aforementioned CVDs, respectively. Finally, updates on E2 therapies for maintaining cardiac health during menopause and suggestions for the advancement treatments were highlighted.

## Introduction

Cardiovascular diseases (CVDs) remain a leading cause of death globally; hence, it is imperative to understand the underlying mechanisms of its pathogenesis. Studies over the decades and clinical demographics have demonstrated the existence of sex–gender disparity in patients developing CVDs. The cardioprotective role of estrogen has been implicated as a factor causing the sex–gender differences in the occurrence of CVDs. This is owing to the fact that the incidence of hypertension, coronary heart disease (CHD), and pathological cardiac hypertrophy are noticeably less in premenopausal women compared with their age cohort men, until the onset of menopause (1).

Besides catecholamine stress, heredity, ethnic background, aging, and lifestyles (smoking, physical inactivity, and obesity) are the traditional risk factors that broadly influences the pathogenesis of CVDs (2).

Under physiological state, circulating catecholamines stimulate the β-adrenergic receptors (βARs) expressed in the heart to regulate inotropic and chronotropic functions of the heart. However, overstimulation of the βARs during chronic catecholamine stress (CCS) dysregulates the receptors (3, 4). Without any timely treatment intervention to halt the excessive firing of the sympathetic–adrenal medullary system or adaptively modulate effectors post-βARs, the receptor dysregulation cascade initiates the pathogenesis and progression of the aforementioned CVDs. Among the subtypes of βARs, the β_2_ARs are mostly found mediating catecholamine stimuli during CVDs (4). The loss of estrogen and its residual cardioprotective effect on preventing the dysregulation of the βARs during CCS predisposes post-menopausal women to developing these CVDs just as men of all age cohorts.

This review from a pathomechanical perspective aims at illustrating the roles of E2 in the modulation of stimuli signaling in the heart during CCS and also highlights its implication in causing the obvious sex–gender differences in CVD patients. We initially introduced the subtypes, localizations, and functions of ERs and βARs in the heart briefly. The signaling cascades via ERs and βARs under physiological state and CCS state were summarized. In addition, an overview of how E2 deficiency permits the signaling cascade that dysregulates βARs to initiate and facilitate the progression of CVDs during CCS was discussed. The prior discussions were then reconciled to explain the following: first, how E2 deficiency contributes to the disruption of nitric oxide (NO) synthesis, thereby dysregulating vasoconstriction and vasodilation, which results into hypertension and arrhythmias during CCS; second, dysregulation of lipid profiles due to E2 deficiency, which causes atherosclerosis and progresses into CHD; and lastly, how E2 deficiency permits the upregulation of nuclear factor of activated T cells (NFATs), thereby predisposing post-menopausal women to the occurrence of PCH during CCS. Furthermore, updates on E2 therapies for maintaining cardiac health during menopause and suggestions for the advancement treatments were highlighted.

## ERs and βARs Interplay in the Cardiovascular System

ERs could be broadly categorized as non-genomic and genomic, based on both their localization and functions. ERα and ERβ are the classical genomic ERs (Table 1). In the myocardium, they mediate the non-rapid estrogenic activation of transcriptional factors (TFs), such as the signal transducer and activator of transcription (STAT) family, CCAAT-enhancer-binding protein beta (C/EBPβ), CREB, Elk-1, and the nuclear factor kappa B (NF-κB) complex (5, 29–31), while G-protein-coupled estrogen receptor (GPR30) is predominantly cytomembrane bound but also expressed on the endoplasmic reticulum in cardiomyocytes. GPR30 mediates rapidly the non-genomic effects of E2, such as influencing ion channel activities. Besides mediating non-genomic estrogenic effects, GPR30 can indirectly activate TFs via interactions with ERα and ERβ, as reviewed extensively here (5). Intriguingly, inhibition of the non-genomic signaling impeded transcriptional response of genes involved in the cardiovascular function (9). Under physiological state, GPR30 signals to enhanced cardiomyocyte inotropy (rapid response) via G_α*s*_/cAMP/PKA/EPAC pathway, but it switches the signaling via G_α*i*_/PI3K/Akt pathway to exert anti-inotropy and antiapoptosis during CCS (10). By the latter, estrogen confers cardioprotection through GPR30 during CCS. In addition, stimulating protein-1 (Sp-1), found downstream the estrogenic pathway, is a crucial mediator of the indirect genomic signaling that regulates the expression of endothelial nitric oxide synthase (eNOS) and low-density lipoprotein (LDL) receptors (LDLRs) (5). LDLR regulates circulation of cholesterol levels, while NOS facilitates vasodilation, vasoconstriction, and anti-inflammatory responses under normal physiological conditions (32, 33). These are crucial homeostatic roles played by E2 and ERs in ensuring proper cardiovascular function under normal physiological states.

**Table 1:** Characteristics and activities of estrogen receptors (ERs) and β-adrenergic receptors (βARs).

**Receptors**	**GPR30**	**ERα**	**ERβ**	**B_**1**_AR**	**β_2_AR**	**B_**3**_AR**	**References**
Localization	Cytosol, plasma membrane, endoplasmic reticulum	Nucleus, cytosol, plasma membrane	Nucleus, cytosol, plasma membrane	Plasma membrane	Plasma membrane	Plasma membrane	(5–8)
Gene transcription regulation	Non-genomic, Indirect genomic	Genomic	Genomic	Non-genomic	Non-genomic	Non-genomic	(5, 9)
Agonist specificity	E2, G-1	E2, PPT	E2, DPN	Epi (<), NE (>), ISO (=), DA (=), DB (>),	Epi, NE, ISO, DA, DB,	Epi, NE, ISO	(10–16)
Pathways	G_α*s*_/G_α*i*_	PI3K/AKT	PI3K	G_α*s*_	G_α*s*_/G_α*i*_/G-protein independent (non-canonically)	G_α*s*_/G_α*i*_	(10, 17)
Physiological effect	Rapid actions (response within seconds to minutes, but indirectly genomic effects are delayed),	Rapid and genomic action (effects induced within minutes to days)	Rapid and genomic action (effects induced within minutes to days)	Rapid	Rapid	Rapid	(5, 9, 18, 19)
Cardiovascular tissue distribution	Cardiomyocytes, fibroblast, vascular tissues	Cardiomyocytes, fibroblast, vascular tissues	fibroblast, vascular tissues	Cardiomyocytes, fibroblast, vascular tissues	Cardiomyocytes, fibroblast, vascular tissues	Cardiomyocytes, adipocytes	(10, 13, 20–22)
Relative expression in cardiomyocytes	Abundant	Less abundant	Least abundant	Abundant in entire myocardium	Abundant in apical myocardium	Least abundant n entire myocardium	(10, 13, 21, 23, 24)
Impact of Estrogen on expression in CVS	Upregulation	Upregulation	Upregulation	Upregulation	Upregulation	Upregulation	(10, 25, 26)
Implication in CVD pathogenesis/complication	~	~	~	Depletion permits HF	Mediation of stress-related cardiomyopathies	Mediates negative inotropy effect, facilitating HF	(4, 24, 27, 28)

*HF, heart failure; CVD, cardiovascular diseases; CVS, cardiovascular system; PPT, propylpyrazoletriol; DPN, propylpyrazoletriol; G-1, GPR30 agonist; Epi, epinephrine; NE, norepinephrine; ISO, isoproterenol; DA, dopamine; DB, dobutamine; <, less affinity compared to β_2_AR; >, higher affinity compared to β_2_AR; =, equal affinity with β_2_AR*.

Just like ERs, βARs are seven transmembrane-spanning receptors; even so, they respond to different agonists and are also well-expressed in the heart (10). βAR subunits β_1_AR and β_2_AR are distinctively expressed in the heart. β_1_AR is widely expressed in all parts of the heart, while β_2_AR is sparingly expressed in the apical myocardium (Table 1). However, β_3_AR is mostly expressed in adipocytes and mediates energy metabolism signals (20). In a normal physiological state, stimulation of β_1_AR and β_2_AR by circulating catecholamines permits signaling via the classical G_α*s*_/cAMP/PKA, just as E2 does on binding to GPR30 (10). This signaling cascade phosphorylates the L-type Ca^2+^ channel to ensure the rhythmic contraction of the myocardium. Under stress condition, β_2_AR, due to its pleiotropic nature, can traffic signaling via G_α*i*_/PI3K/Akt to prevent any cardiac insults. However, during CCS, β_2_AR functions are dysregulated and are also found to be relatively highly expressed in the myocardium than β_1_AR (4, 10). This implicates β_2_AR in the mediation of signaling cascades that initiates the pathogenesis of CVDs and results in heart failures (HFs) (4).

The sharing of similar pathways, G_α*s*_/cAMP/PKA and G_α*i*_/PI3K/Akt by βARs and GPR30 in the cardiovascular system, suggests an interplay between these receptors. A mounting of evidence of the last decades also to prove this (10, 34–36). Besides, even though βARs are not receptors of E2, findings from our recent studies along with others have shown that E2 is able to upregulate the expressions of βARs in cardiomyocytes under stress state and confers cardioprotection by doing so (34, 36). While the underlying mechanisms are still being elucidated, we speculate that E2 via its non-genomic signaling may be modulating activities of G-protein-coupled receptor kinase (GRK) 2 to minimize the internalization of β_2_ARs during stress.

## Estrogen Deficiency and βAR Overstimulation During CCS

E2, being the primary female sex hormones, exerts and modulates several adaptive responses, such as cardioprotection and immunoregulation via its non-genomic and genomic signaling (37). E2 does all these besides its reproductive functions. By the adaptive effects of E2, women have a lower risk of developing CVDs and its associated complications in their reproductive age. The lower risk of CVDs in premenopausal women could be attributed to E2 maintaining the blood pressure (BP) through indirect regulation of lipid profile via LDLR and enhancing NOS activities, preventing cardiac insult during stress via both direct and indirect signaling through Gαi/PI3K/Akt, and conferring vasoprotection and timely regulation of pro- and anti-inflammatory response (37, 38). Unfortunately, male individuals of all age cohorts do not have these estrogenic advantages, as they are predisposed to the occurrence of CVDs. Nonetheless, the deficiency of E2 and the loss of its residual protective effects during menopausal increases the incidence of CVDs just as in men.

**Figure 1:**
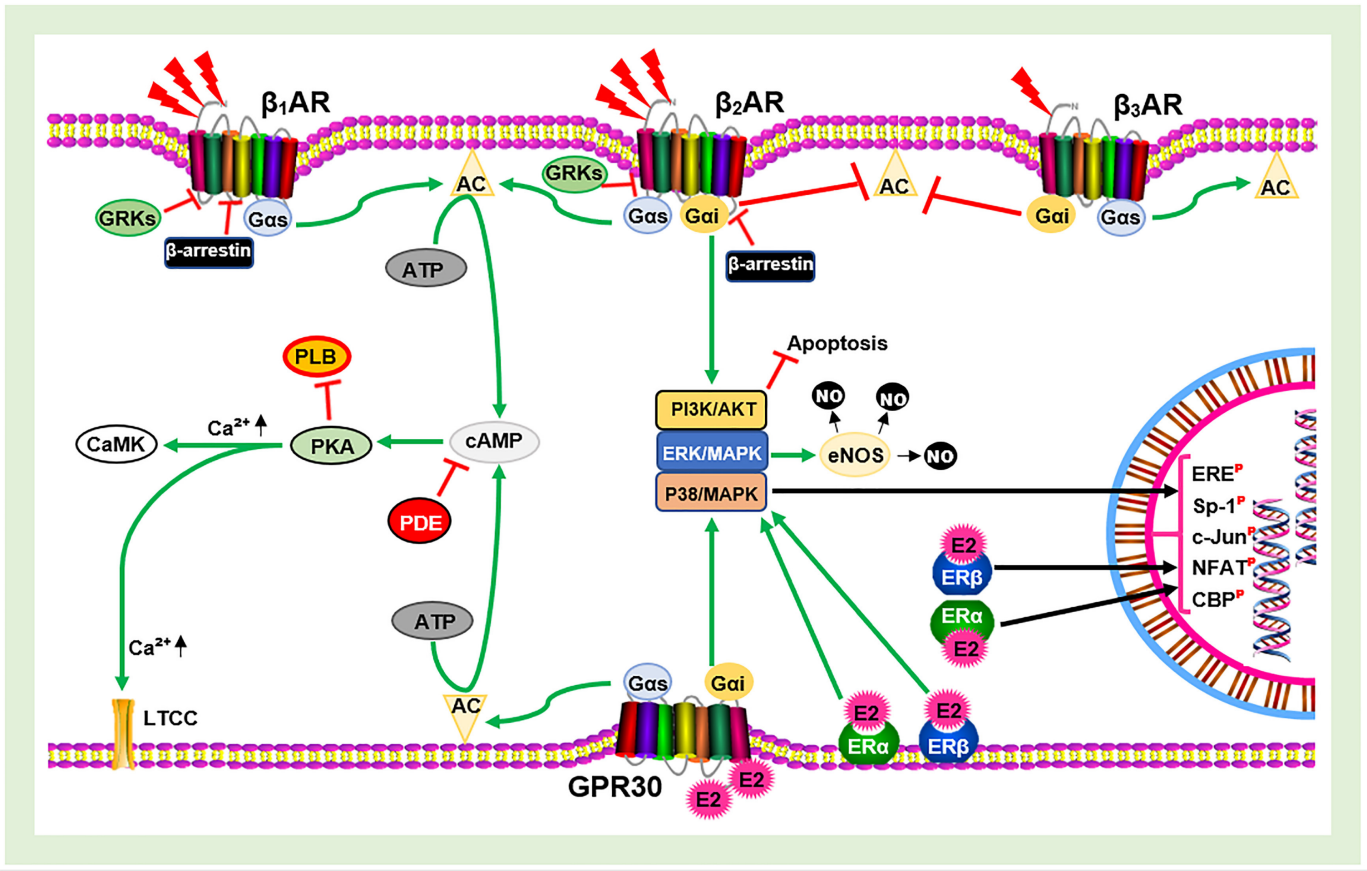
Schematic illustration of estrogen receptors (ERs) and β-adrenergic receptors (βARs) signaling. AC, Adenylyl cyclase; cAMP, cyclic adenosine monophosphate; PKA, protein kinase A; GRKs, G-protein-coupled receptor kinases; E2, estrogen; eNOS, endothelial nitric oxide synthase; NO, nitric oxide; PDE, phosphodiesterase; PLB, phospholamban; LTCC, L-type calcium channel; CaMK, calmodulin-dependent kinase; ERE, estrogen receptor element; Sp-1, stimulating protein-1; NFAT, nuclear factor of activated T cells; 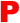, Phosphorylated; 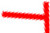, Inhibit; 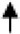, Upregulate; 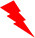, Catecholamine.

At this point, overstimulation of βARs by circulating catecholamines can lead to devastating consequences on the cardiovascular system (CVS). Overstimulation of βARs dysregulates the receptor, usually resulting in the desensitization, internalization, and downregulation of the receptor. This cascade is initiated by PKA negative feedback (heterologous desensitization) or GRK2 phosphorylation—β-arrestins-1 binding at serine/threonine of the receptor (homologous desensitization). These ultimately affects cardiac function and initiates CVD pathogenesis when it prolongs. However, the presence of E2 is able the mitigate the dysregulation of βARs likely via the modulation of GRK2 activities as speculated (39). In addition, E2 signaling via GPR30/G_α*i*_/PI3K/Akt during stress might compliment the β_2_AR/G_α*i*_/PI3K/Akt signaling to enforce the prevention of any cardiac insult (Figure 1). All these adaptive effects of E2 are lost during its deficiency. As such, the adverse effects of βAR hypersensitization during CCS are aggravated in the absence of E2. This is a probable explanation to why women are resilient to the adverse effects of stress on the CVS until the onset of menopause.

## Mechanistic Roles of Estrogen in Catecholamine Stress-Related Sex-Gender Disparities in CVDs

Herein, we discuss the probable signaling cascades facilitated by E2 to prevent the occurrence of hypertension, coronary heart disease, and pathological cardiac hypertrophy. Although there are many other CVDs occurring due to CCS and have sex–gender disparities, the focus of this review will be centered on these CVDs.

### Hypertension

Hypertension is a constant increase in BP that causes damage to several organs. The pathophysiology of hypertension comprises arrhythmias, elevated systemic vascular resistance, stiffness, and receptivity to stimuli, which can result in other CVS-related pathological conditions (40). Vasodilation and vasoconstriction are the opposing physiological functions that regulate the tension of blood in circulation. They are essential for maintaining homeostatic function as they retain body heat, minimize blood loss during injuries, and regulate of arterial pressure. Vasodilation is facilitated by the activities of nitric oxide (NO); as such, the bioavailability of NO is crucial for the regulation of the tension/pressure of blood in circulation. The eNOS produces the NO explicitly in the vascular endothelium to induce the vasorelaxation (41). The impairment of eNOS and deficiency of NO leads to endothelial dysfunction, prolonged vasoconstriction, and an increase in BP (41). Prolongation of these vascular dysfunctions ultimately results in hypertension.

In a Ca^2+^-independent manner, E2 and shear stress (circulating catecholamine) are the known agonists that activate eNOS. By signaling via GPR30/G_α*i*_, E2 induces Akt, ERK1/2, or CaMK-II to phosphorylate eNOS at Ser1177, thereby activating eNOS and enhancing NO production for normal vascular function (42, 43). In addition, stimulation of β_2_AR by catecholamines induces NO production via G_α*i*_/PI3K/Akt phosphorylation of eNOS (44). However, during CCS, β_2_AR dysregulation and dysfunction may impede its mediation of the activation of eNOS, as such affects the NO bioavailability and causes endothelial dysfunction. In premenopausal women, the inability of β_2_AR to facilitate NO production might be masked by E2-induced activation of eNOS–NO production. Therefore, the occurrence of catecholamine-stress-induced endothelial dysfunction, which progresses in hypertension, may be averted by E2 in such age cohort of female individuals. Besides, E2 also prevents the dysregulation of β_2_AR; hence, the adverse effect of CCS on NO production may be mitigated by it. Nonetheless, the deficiency E2 and the loss of its residual protective effects (typically during menopause) permit endothelial dysfunction, and its follow-up cascades that result in hypertension and HF. Unfortunately, male individuals of all age cohort are pre-disposed to developing HF resulting from hypertension during CCS (45).

Furthermore, it has been suggested that E2 exerts the following adaptive effects on the renin–angiotensin system (RAS) pathway to prevent hypertension: (1) E2 enhances signaling of angiotensin II (Ang II) via angiotensin II receptor type 2 (AT_2_R) to facilitate vasodilation (2, 46) E2 upregulates angiotensin-converting enzymes (ACE) 2 expression to enhance the conversion of Ang II to Ang-(1–4, 29–31) for signaling via Mas receptor (MasR) to facilitate vasodilation (47). Detailed mechanistic role of E2 in preventing hypertension via the RAS pathway has been demonstrated elsewhere (48, 49).

### Coronary Heart Disease

CHD is the ultimate result of coronary artery disease (CAD), although these CVD terms are used interchangeably. CAD is characterized by a reduction in blood circulation to the myocardium caused by obstructive atherosclerotic (fibro-fatty plaque buildups) vasoconstriction in the epicardial coronary arteries (50, 51). The prolonged reduction in blood flow to the myocardium (ischemia) causes heart attacks or induce cardiomyocytes death and myocardial infarction, which elicits inflammatory responses and consequently marked fibrosis (51). The fundamental pathomechanism of CHD initiation and progression has to do with lipid accumulation in the CVS. Moreover, unhealthy lifestyles, such as smoking and obesity [high low-density lipoprotein (LDL) cholesterol, low high-density lipoprotein (HDL) cholesterol], aging, hypertension, family history of premature atherosclerosis, hyperlipidemia, diabetes, chronic stress, and menopause are its known risk factors (45). Among these traditional risk factors, chronic stress and E2-deficiency-related factor will be the focus of this study.

NO made available due to shear stress signaling via β_2_AR contributes to the retardation of atherosclerosis, besides regulating blood pressure. However, CCS is implicated in the pathogenesis of CHD because it dysregulates β_2_AR (44) and induces dysfunction of eNOS, thereby expediting the occurrence of atherosclerosis. In addition, compared to premenopausal women, post-menopausal women are at an increased risk of developing CAD/CHD due to loss of the pre-discussed vasodilation and vasoprotective roles E2 (42, 43).

Nonetheless, since CHD pathogenesis is involved in lipid accumulation in the intimal layer of arteries, this suggests that besides vasoregulation, E2 and ERs may be using multiple mechanisms to regulate lipid profile levels in circulation and impeding LDL depositions to minimize the occurrence of CHD premenopausal women. Efforts to elucidate the mechanisms employed by E2 and ERs to result in the sex–gender differences in the development of CHD have suggested the following.

First, physiological levels E2 signaling via ERα upregulates the expression of LDLR and downregulates scavenger receptor class B type 1 (SR-B1), the HDL receptor (52). By this, E2 enhances rapidly the uptake of LDL (bad cholesterol) in circulation while keeping circulating levels of HDL (good cholesterol) high, thus keeping the lipid profile in a healthy balance. Unlike HDL, which gets to the liver and helps with the removal of other forms of cholesterol from the bloodstream, LDL only gets deposited in arterial walls (atherosclerosis); thus, they are given the names good and bad cholesterol, respectively. In short, these studies have demonstrated that E2 via ERα upregulated LDLR expressions to decrease bloodstream levels as well as the deposition of LDL to prevents CAD/CHD (52, 53).

In addition, Fu et al. recently demonstrated that by non-genomic signaling via GPR30-phospholipase C-γ (PLCγ), E2 preserves the expression of LDLR (54). Proprotein convertase subtilisin/kexin type 9 (PCSK9), a negative modulator of lipid metabolism, has been shown to mediate the degradation of LDLR. PCSK9 does this by binding to the LDLR extracellular domain and then facilitating LDLR endocytosis and degradation by lysosomes (55). By degrading LDLR, PCSK9 elevates circulating LDL levels and increases the risk of CAD/CHD. However, E2 via GPR30 was shown to inhibit the PCSK9-mediated cascade that degrades LDLR, thereby preserving LDLR expressions and keeping bloodstream LDL levels and the risk of CAD/CHD low (54).

Furthermore, it has been suggested that besides downregulating SR-B1 to keep bloodstream HDL levels high via ERα signaling, E2 also inhibits LDL transcytosis via GPR30 and SR-B1 (56). Transcytosis is an integral vesicular that helps transport macromolecules (e.g., LDL) across the interior of a cell by endocytosis of the molecule at the apical endothelial surface and exocytosis at the basal membrane. Intriguingly, SR-B1 was identified as one of the receptors that can transport LDL through endothelial cells of the human coronary artery (57). However, the finding of Ghaffari et al. demonstrated that instead of ERα, GPR30 was specifically found mediating the downregulation of SR-B1 and the inhibition of LDL transcytosis to lower the rate of atherosclerosis (56) and ultimately ischemia and CAD/CHD.

Lastly, based on the well-known adaptive immunoregulatory roles of E2 and its receptors (38), it can be speculated that in cases where there are sudden ruptures of atherosclerotic plaque, E2 signaling via ERs may lessen the platelet aggregation and thrombosis while modulating timely pro- and anti-inflammatory responses to mitigate the possible fatal outcomes. Conversely, androgen has been demonstrated to facilitate maladaptive and immunosuppressive responses (58–63). This is evident, as data over the decades have suggested that more men and post-menopausal women die from atherosclerosis/CAD/CHD than premenopausal women (37, 38). In summary, all these findings have shown that deficiency of E2 and dysregulation of ERs (both genomic and non-genomic) distort healthy balance in lipid profiles and permits LDL depositions into the intimal layer of the coronary arteries, which progresses into CHD.

### Pathological Cardiac Hypertrophy

The chronic upregulation of levels of circulating catecholamines overstimulates β_2_AR, inducing a non-canonical signaling that is facilitated by GRK5 phosphorylation—β-arrestins-1 binding at serine/threonine of the receptor (17, 64). In addition, because GRK5 has a nuclear localization sequence (NLS) identical to homeobox-containing transcription factors, it can translocate to the nuclei where it activates the TFs: myocyte enhancer factor 2 (MEF2), GATA4, Csx/Nkx2–5, and NF-kB (17, 65). An irreversible pathological cardiac hypertrophied (PCH) heart is the result of these cascades if there are no timely intervention to subdue the adverse remodeling. PCH patients suffer heart malfunction resulting from markedly thickened ventricular walls and a decrease in the left ventricular chamber. As such, it becomes impossible for the heart to replenish sufficient blood rapidly for the next ejection (66).

In the recent decade, considerable efforts have been devoted to elucidating the pathomechanism underlying the occurrence of PCH during CCS. Besides the maladaptive roles played by GRK5, the nuclear factor of activated T cells, the cytoplasm (NFATc) is at the center of the signaling cascades maladaptively remodeling the heart. Our earlier studies suggested that PCH maybe the result of synergies of maladaptive signal cascades occurring in cardiomyocytes and immune cells; intriguingly, NFATs play essential roles in these cells (4). Of the four intracellular Ca^2+^-dependent isoforms of NFAT (NFATc1–NFATc4) reviewed elsewhere (67), NFATc3 has been shown to be the isoform implicated in facilitating the exacerbation of PCH (17). Under physiological state, NFATs are cytosolic due to being heavily phosphorylated. However, activation of intercellular calcium regulating receptors (e.g., ERs) upregulated Ca^2+^-calmodulin-activated calcineurin, which dephosphorylates the NFATs, enabling them to translocate in the nuclei and regulate gene transcription adaptively (68). Hullmann et al. demonstrated that during chronic stress (pressure overload), GRK5 activated NFATc3 in a kinase-independent manner, which exacerbated PCH (17).

As well-demonstrated, NFATc3 plays an essential maladaptive role in the adverse remodeling of the heart; nonetheless, E2 and ERs have been shown to modulate their activation and signaling activities adaptively to exert antihypertrophic effects. First, E2 has been demonstrated to suppress nuclear localization of GRK5 (69), thereby impeding its activation of NFATc3. In addition, the overexpression of ERα and ERβ were found to have repressed the transcriptional activities of NFATc3, while the contrast was observed when endogenous ERα and ERβ were knocked down. Furthermore, an agonist of ERα (delphinidin) was found to inhibit NFAT activation and also inhibited histone deacetylase (HDAC), which also has the ability to participate in inducing PCH (70, 71). This suggests that even though E2 increases intracellular Ca^2+^ concentrations to modulate NFATc3 activities via non-genomic signaling (GPR30) adaptively, it might also have the ability to repress or normalize intracellular Ca^2+^ concentration via genomic signaling (ERα and ERβ) to inhibit NFATc3 activities and attenuate the occurrence of PCH during CCS. Finally, maladaptive inflammatory responses are also implicated in the pathogenesis and exacerbation of PCH (4); however, the adaptive immunoregulatory roles of E2 might mitigate the extent of adverse cardiac remodeling, while androgen does the contrary. As such, the deficiency of E2 and/or depletion in ER expression predisposes an individual to the occurrence of PCH during CCS.

Altogether, these might also explain why premenopausal women have a low tendency of developing PCH, while male individuals of all age cohorts and menopausal women are likely to have their hearts pathological remodeled when having CCS.

## Conclusions and Perspectives on E2 Replacement Therapy

**Figure 2:**
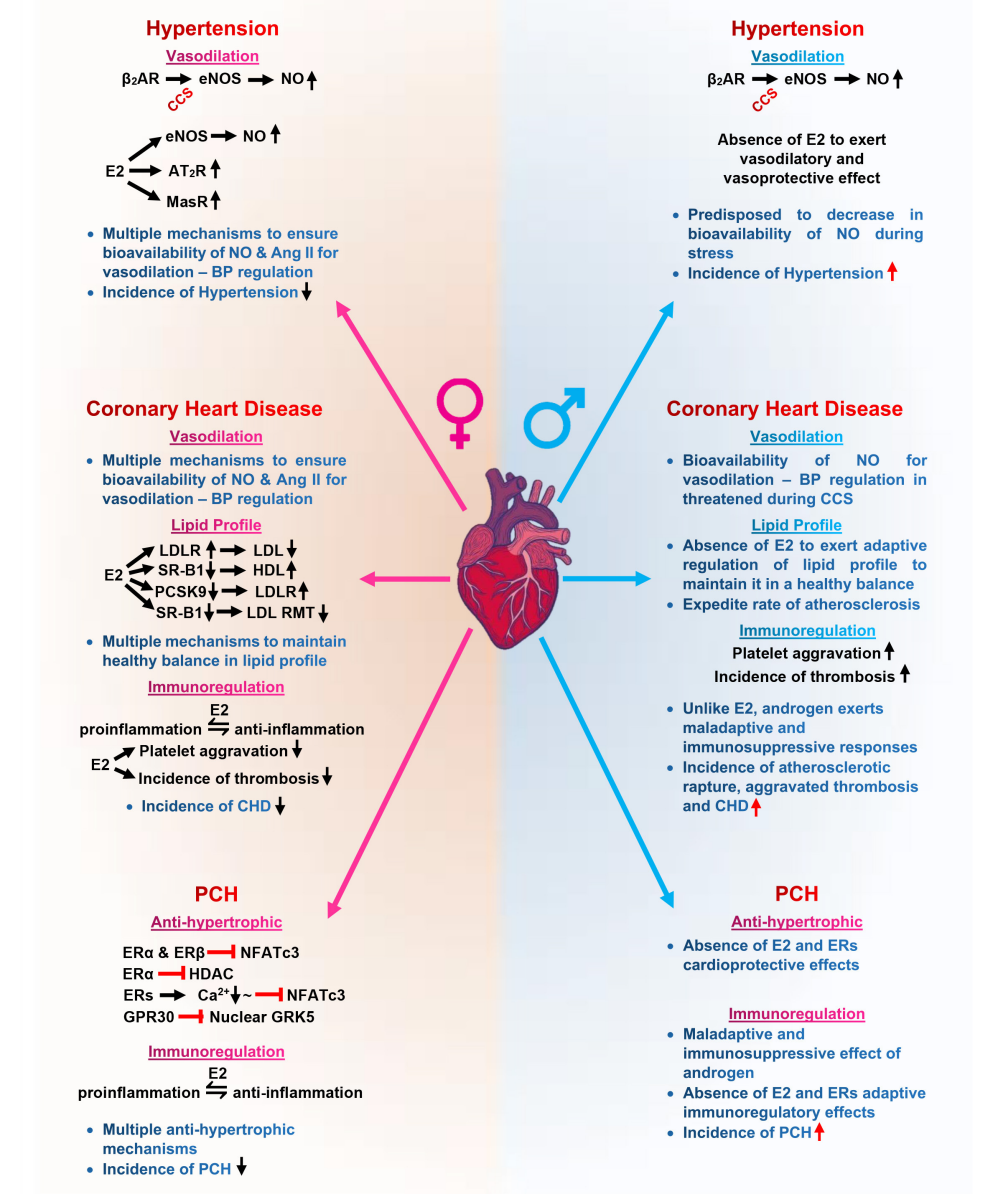
Graphical abstract of sex–gender disparities in cardiovascular diseases mediated by the mechanistic role of estrogen on eNOS, lipid profile, and NFATs. eNOS, endothelial nitric oxide synthase; NO, nitric oxide; Ang II, angiotensin II; AT2R, angiotensin II receptor type 2; MasR, Mas receptor; LDL, low-density lipoprotein; LDLR, low-density lipoprotein receptor; RMT, receptor-mediated transcytosis; SR-B1, scavenger receptor class B type 1; PCSK9, proprotein convertase subtilisin/kexin type 9; GRK5, G protein-coupled receptor kinases 5; HDAC, histone deacetylase; 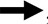, signaling; 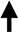, upregulate; 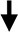, downregulate; 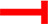, inhibit; 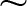, normalize.

Sex–gender disparities in the clinical demographics of hypertension, CAD/CHD, and PCH have been demonstrated to be likely due to the diverse protective mechanistic roles of E2 and ERs in modulating signaling cascades even during CCS, to circumvent adverse outcomes in premenopausal women (Figure 2). In contrast, male individuals of all age cohorts are disadvantaged due to lack of physiological levels of E2. The incidence rates of these CVDs are higher in male individuals, and their lack of estrogen expedites the progression into heart failure and death.

E2 replacement therapy (E2RT) is used to control menopausal symptoms and minimize the incidence of cardiovascular disease in post-menopausal women (53, 72, 73). However, the usage of E2RT decreased significantly, following reports from the randomized controlled trial by the Women's Health Initiative (WHI), which concluded that an increased risk of breast cancer was associated with using menopausal hormone therapy (MHT) (74–79). Attempts to explain the finding of WHI revealed that the type and regimen of MHT used during the randomized controlled trial might have influenced the increased risk of breast cancer. In summary, the combination of conjugated equine estrogen plus medroxyprogesterone acetate has been advised against (80–82). In addition, it has been suggested that the MHT should be initiated early within the critical window (5–6 years from menopausal) to circumvent the reported side effects of MHT (83, 84). The recent findings of Shao et al. and Hodia et al. have provided evidence to support the need to initiate MHT within 5–6 years after menopause (83, 85). It was demonstrated that individuals who started oral E2RT within 6 years after menopause had less progression of atherosclerosis than those who started 10 years or more after menopause (83).

Furthermore, it has also been demonstrated that by targeting GPR30, post-menopausal coronary atherosclerosis and its accompanying maladaptive inflammatory responses were attenuated without any side effect. It was explained that the agonist (G-1) via GPR30 did not have any uterotrophic effects; hence, no malignancies and hyperplasia were observed (86). Based on this evidence, it is proposed that by initiating E2RT within 5–6 years after menopausal or by exclusively targeting GPR30, the incidence of hypertension, CHD, and PCH may be minimized in post-menopausal women with fewer side effects.

## Author Contributions

The review idea was conceived by MN. GA, MN, and RM drafted and wrote the manuscript. With the supervision of HS, GA, MN, RM, AA, and JA-A revised and proofread the manuscript. All authors accepted the final version of the manuscript.

## Conflict of Interest

The authors declare that the research was conducted in the absence of any commercial or financial relationships that could be construed as a potential conflict of interest.
